# The Human Microbiome as a Therapeutic Target for Metabolic Diseases

**DOI:** 10.3390/nu16142322

**Published:** 2024-07-19

**Authors:** Thi Phuong Nam Bui

**Affiliations:** Department of Experimental Vascular Medicine, Amsterdam University Medical Center, 1105 AZ Amsterdam, The Netherlands; t.p.n.bui@amsterdamumc.nl

**Keywords:** human microbiota, host metabolism, microbial metabolites, short chain fatty acid, bile acid, metabolic disease

## Abstract

The human microbiome functions as a separate organ in a symbiotic relationship with the host. Disruption of this host–microbe symbiosis can lead to serious health problems. Modifications to the composition and function of the microbiome have been linked to changes in host metabolic outcomes. Industrial lifestyles with high consumption of processed foods, alcoholic beverages and antibiotic use have significantly altered the gut microbiome in unfavorable ways. Therefore, understanding the causal relationship between the human microbiome and host metabolism will provide important insights into how we can better intervene in metabolic health. In this review, I will discuss the potential use of the human microbiome as a therapeutic target to improve host metabolism.

## 1. Introduction

The human microbiome resides in our gastrointestinal tract and creates a dynamic and complex microbial ecosystem consisting of more than 1000 microbial species [1] and their phages. Based on epidemiological and omics studies combined with in vitro studies using various cell models and in vivo studies in mice, it appears that human health and disease risk may be mediated by the human microbiome [2]. In infancy, the composition and function of the human microbiome are influenced by the mode of birth and feeding, and it becomes stable by the age of three [3]. In adult life, these microbes are mainly influenced by lifestyle, medication and host genetics [4]. The gut microbiota, in turn, produce microbial components that act not only on local cells in the gut but also on peripheral tissues via the systemic circulation, playing a crucial role in training our immune system and regulating gut endocrine function and neurological signaling [2,5]. They are also involved in modifying drug action and metabolism, eliminating toxins and producing numerous signaling compounds. From time to time, we learn more about new functions of the gut microbiome.

There is an increasing global prevalence of metabolic diseases associated with unhealthy lifestyles [6]. These include type 2 diabetes (T2D), metabolic dysfunction-associated steatotic liver disease (MASLD), hypertension, hyperlipidemia and obesity. In 2019, the world was estimated to have around 44 million cases of T2D and 1.2 billion cases of MASLD [6], and these numbers are increasing rapidly. This rapid increase has been attributed to the overuse of processed foods, urbanization, smoking addiction and physical inactivity, resulting in a significant increase in people with poor metabolic health [7,8,9]. Despite the wide variation in the pathologies of these common metabolic disorders, they are all associated with abnormalities in the composition and function of the human microbiota [10,11,12]. It remains questionable whether there is a causal relationship between host metabolism and the microbiome. To date, results obtained from animal and fecal microbiota transplantation studies have demonstrated causal effects of the microbiome on host health [13,14,15]. Importantly, recent developments in next-generation microbiome sequencing to obtain comprehensive gene catalogues combined with targeted bioinformatics have provided a substantial amount of new knowledge on the role of the gut microbiota in human metabolism. Here, the implications of the human microbiome as a therapeutic target for metabolic disease will be discussed.

## 2. Interplay between the Human Microbiota, Diet and Health Determines Metabolic Outcome

**Figure 1:**
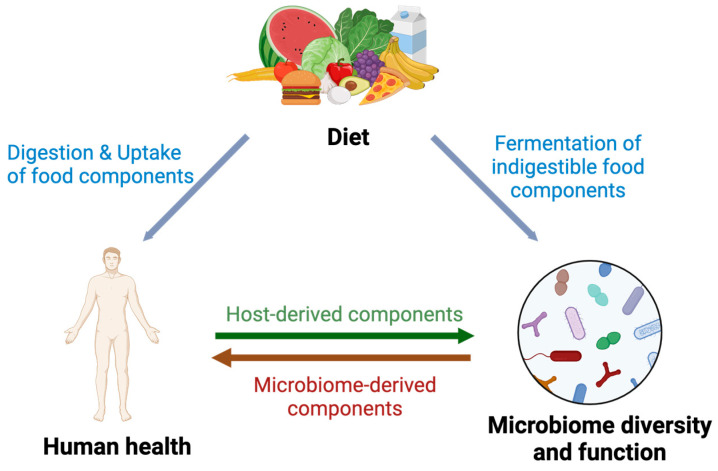
Complex interplay between the gut microbiome, diet and host health. Diet provides nutrients that are absorbed directly by the host and metabolized by the microbiome via fermentation, resulting in the production of metabolites that affect the host. In turn, the host provides its own components to the microbiome, such as mucin and mucus-derived glycans.

The gut microbiome has been shown to play a critical role in gut homeostasis and beyond. Several cohort studies have reported profound associations between the human microbiome and host health [2,16,17]. These associations are strongly influenced by environmental factors, with diet being one of the most dominant drivers [18], creating a complex interplay between the human microbiome, diet and the host that determines the health outcomes (Figure 1). Diet provides nutrients to both the host and the microbiome through the digestion and ingestion of food components, and the microbial fermentation of indigestible food components, respectively. More importantly, there is considerable crosstalk between the host and the microbiome, with the host producing components for microbial activities such as mucin, whereas the microbiome uses both host-derived and dietary components not only for growth but also for the production of components that affect host health. Thus, diet shapes the microbiome composition and metabolic activities navigating towards either beneficial or detrimental effects [19], and these effects, in turn, are strongly mediated by the human microbiome [20].

Consuming a healthy diet, such as one rich in fiber, benefits our health by modulating the human microbiome to produce beneficial compounds such as short chain fatty acids [21]. Moreover, fiber has been shown to improve bowel function as well as intestinal function and transit time [22], thereby increasing microbial metabolism and mucosal turnover [23]. Similarly, low microbiome gene richness and clinical phenotypes have been increased by dietary intervention in obese and overweight individuals [18]. A recent human study showed that supplementation with fermented foods reduced markers of inflammation, and high-fiber dietary intake increased microbiota carbohydrate active enzymes [20]. Surprisingly, increased pro-inflammatory cytokines were observed in subjects with low baseline microbial diversity in the high-fiber diet group. This is an interesting observation as it is generally accepted that fiber intake improves host health via bacterial fermentation to produce short chain fatty acids, signaling molecules with important metabolic functions [21]. It may be necessary to increase the microbial diversity in the gut prior to fiber ingestion to achieve health benefits. Not only the type of food intake but also the eating pattern affect the microbiome. Intermittent fasting has been shown to modulate the gut microbiota and improve obesity and host energy metabolism [24].

In contrast to the dietary benefits of microbiome modulation, there are also adverse effects of unhealthy diets via gut microbial activities; for example, consumption of ultra-processed foods may increase the risk of cardiometabolic disease [25]. Diets that are largely heat-processed contain substantial amounts of advanced glycation end products, which are the result of a cross-linking non-enzymatic reaction between reducing sugars and free amino groups of proteins, nucleic acids or lipids. Dietary advanced glycation end products have been shown to contribute to increased inflammation, oxidative stress and modulation of insulin sensitivity in overweight individuals [26]. The excessive consumption of refined sugars has been correlated with adverse effects on the gut microbiome, including reduction in signaling molecules (short chain fatty acids), altered gut barrier function and increased inflammation, leading to various metabolic disorders [27]. In fact, it has been shown that ethanol derived from the human microbiome activities contributed to the development of MASLD [28]. In a human study, artificial sweeteners such as sorbitol were found to induce glucose intolerance by altering the gut microbiota [29]. It has become clear that the balance between the microbiome, the diet and the host is important for maintaining host health. Once this balance is disturbed, changes in both the microbiome and diet may be required to achieve optimal health benefits.

## 3. Altered Microbiota in Metabolic Syndrome

**Figure 2:**
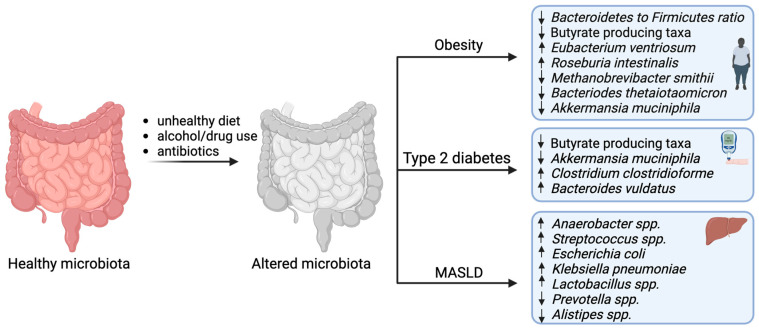
Altered gut microbiota in metabolic disease. Unhealthy diet, alcohol or drug use and antibiotics alter the composition and function of the microbiota in unfavorable ways, which may contribute to the development of metabolic disorders. Despite the wide variation in the pathologies of chronic metabolic disorders, a few microbial groups are commonly reduced in individuals with metabolic disorders such as butyrate-producing taxa and *Akkermansia muciniphila*. These bacterial species may therefore be potential therapeutic targets for the treatment and prevention of metabolic disorders. Down arrow as reduction and up arrow as increase.

A healthy microbiome is characterized by a high diversity of microbial taxa, high microbial gene richness and stable microbial functional cores [17]. Several cohort studies have shown that the microbiome is altered in metabolic diseases as compared to healthy controls (Figure 2). It has been observed that the microbiome of obese individuals differs significantly from that of healthy individuals. It was first suggested that the gut microbiota of obese mice and obese humans had a higher ratio of members of the phylum Firmicutes to members of the phylum Bacteroides compared to that of lean counterparts [30]. In addition, obese individuals had a lower microbial diversity than that of lean individuals, but other studies in humans and rodents have found no difference in this ratio in obese versus lean individuals, and weight loss had no effect on this ratio [31,32,33]. Therefore, the Firmicutes/Bacteroides ratio may not be a good marker of obesity. At the species level, some species were associated with a high BMI, such as *Eubacterium ventriosum* and *Roseburia intestinalis* [34], while other butyrate producers and the methanogenic archaeon *Methanobrevibacter smithii* may be associated with leanness [35]. Another metagenome-wide association showed that the abundance of *Bacteroides thetaiotaomicron* was significantly reduced in obese compared to lean individuals, and the administration of this bacterium protected against obesity in mice [36]. In two independent cohorts, structural variations carrying *myo*-inositol metabolic genes in the *Anaerostipes hadrus* genome showed inverse correlations with body weight and body mass index [37]. It has been shown that obesity can be transmitted from humans to mice via fecal microbiome transplantation in a diet-dependent manner [38], suggesting opportunities to intervene in whole-body energy metabolism by targeting the microbiome.

Like obesity, T2D is one of the most common endocrine disorders with increasing prevalence and incidence, affecting nearly 15% of the adult population [39]. The etiology of T2D involves multiple genetic and environmental factors, including the human microbiome. The association between the human microbiome and type 2 diabetes was first reported in 2012 [10]. Following this study, the richness of the human microbiome was found to correlate with several metabolic biomarkers [40]. Many cross-sectional studies have shown that the microbiome of T2D subjects has reduced microbial gene diversity, with a decrease in butyrate-producing species and *Akkermansia muciniphila*, and an increase in bacterial strains with pro-inflammatory functional potential [10,41]. To date, a few bacterial strains have been found to improve insulin sensitivity and glycemic variability in metabolic syndrome, obesity and T2D [13,42,43]. Metformin, a commonly used drug for diabetes, has been shown to favorably alter the gut microbiome, contributing to its therapeutic effects [44]. Potential bacterial candidates that influence metformin therapy have been identified using a host–microbe–drug–nutrient screen [45]. Statin therapy has been found to be associated with a reduced prevalence of gut microbiota dysbiosis [46]. Future research is needed to determine whether there are synergistic effects of conventional drugs and microbiome-based therapeutics or multiple strains inT2D.

Recent research has suggested a role for the human microbiome in the pathogenesis of MASLD [47], and there is growing evidence of a perturbed microbiome in MASLD patients, with increased abundance of *Anaerobacter*, *Streptococcus*, *Escherichia* and *Lactobacillus* and decreased abundance of *Prevotella* and *Alistipes* as compared to healthy controls [48]. It has been shown that the microbiota of MASLD patients had an enrichment of *Lactobacillus*, which is responsible for endogenous ethanol production and contributes to the pathogenesis of the disease [28]. Indeed, it has been described that ethanol activates nuclear factor-kB (NF-kB) signaling pathways and impairs intestinal barrier function [49], potentially causing oxidative damage to hepatocytes, which may induce hepatic inflammation. Supplementation with an ethanol-producing strain accelerated MASLD pathogenesis in mice [50]. It remains unknown whether there are gut bacteria that can consume ethanol and whether supplementation with these ethanol-consuming strains can reduce endogenous ethanol production and thereby prevent the progression of MASLD.

## 4. The Human Microbiome as Therapeutic Target to Improve Metabolic Health

Accumulating evidence from both humans and rodents supports the hypothesis that low-grade, systemic and chronic inflammation induced by the gut microbiota may (partially) drive metabolic diseases such as obesity, diabetes and MASLD in humans. This provides the rationale for the ongoing intensive search for gut microbial messengers and gut bacteria that regulate whole-body energy metabolism. Impaired gut barrier function has been implicated in the pathogenesis of several diseases including type 1 diabetes and inflammatory bowel disease (IBD) [51]. Disruption of the gut barrier increases the influx of immunostimulatory microbial ligands into the systemic circulation, potentially leading to obesity, diabetes [52] and MASLD [53]. Gut bacteria that produce components from either dietary or host-derived glycans that enhance gut barrier function and thereby reduce intestinal permeability may serve as a novel therapeutic target for systemic inflammation in metabolic diseases [54]. For example, *Akkermansia muciliphila*, which is abundant in the mucosal lining, functions as a guardian of the gut barrier but also has protective effects against obesity and diabetes [55].

**Figure 3:**
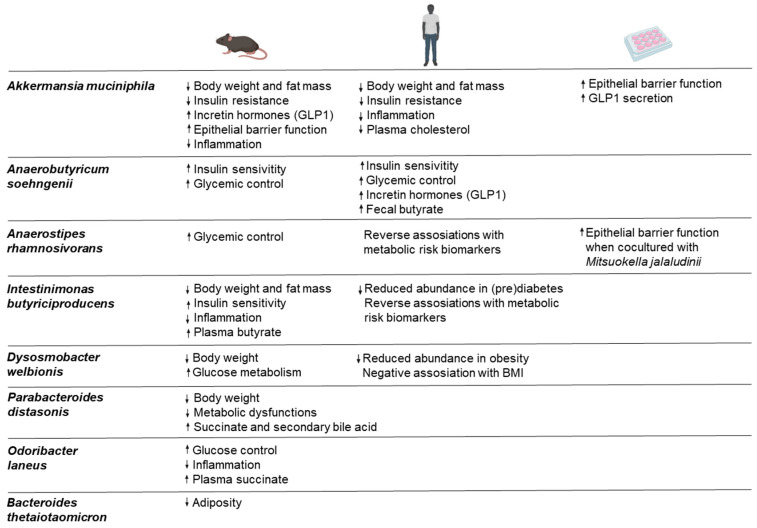
Potential therapeutic gut microbes with proven metabolic health benefits. GLP1: Glucagon-like peptide 1; BMI: Body mass index. Down arrow as reduction and up arrow as increase.

A causal relationship between the gut microbiota and host metabolism was first demonstrated in FMT studies. The gut microbiota composition at baseline was shown to mediate the improvement in insulin sensitivity in FMT-treated metabolic syndrome [56]. This study suggests the microbiome as a mediator of insulin sensitivity regulation. Similar results were obtained when fecal material from lean donors was transferred to subjects with metabolic syndrome, which was associated with increased activities of butyrate-producing bacteria in the small intestine [15]. Gut microbiota and their metabolites have been suggested to modulate host epigenetic alterations in MASLD [57]. A randomized clinical trial showed that FMT treatment delayed the progression of MASLD by altering the gut microbiome, and the beneficial effects were more pronounced in lean MASLD than in obese MASLD individuals [58]. Due to the risk of introducing unwanted components from the fecal samples during FMT treatment [59], efforts have been made to construct synthetic bacterial communities that resemble the healthy microbiome [60]. A mixture of 17 *Clostridium* species induced colonic regulatory T cells in mice [61]. Some success has been achieved in the treatment of *Clostridioides difficile* infection [62,63]. This approach is promising but also presents challenges due to the complexity of the microbial communities that need to be maintained. The fermentation and production of multiple strains for preclinical and clinical studies are other issues. Some encouraging data have been obtained from single bacterial strain treatments to improve metabolic performance in mice and humans (Figure 3). These are discussed in the following sections.

## 5. Potential Therapeutic Bacteria for Metabolic Health

*Akkermansia muciniphila* is a mucin-degrading bacterium that resides abundantly in the mucus layer of mammals [64]. Metagenomic analyses revealed a high phylogenetic and functional diversity of *Akkermansia* in humans [65]. This bacterium is capable of degrading mucin and sugars to produce the short chain fatty acid propionate [66], which can bind to G-protein receptors expressed by L-cells, thereby stimulating GLP-1 secretion. Its unique niche on the mucus layer allows this bacterium to interact directly with the host. *A. muciniphila* has been shown to improve epithelial barrier function via secreted components and outer membrane components, contributing to health-promoting effects [67,68]. Lower levels of *A. muciniphila* have been associated with obesity and diabetes [40,69]. Supplementation with live *A. muciniphila* reversed high-fat-diet-induced metabolic disorders, including body weight gain, adipose tissue inflammation and insulin resistance in mice [69]. Interestingly, anti-obesity and anti-diabetes effects have also been observed with pasteurized *A. muciniphila* and an outer membrane protein Amuc-1100 [67], opening up new therapeutic options to target obesity and diabetes. A proof-of-concept study in obese and overweight individuals showed that daily bacterial administration for 3 months improved insulin sensitivity and reduced insulinemia and plasma cholesterol with a small reduction in body weight [13]. Furthermore, metformin has been reported to increase the relative abundance of *A. muciniphila* in vitro and in humans [44,70], supporting a protective role of this bacterium in metabolic disorders. Taken together, *A. muciniphila* has a great potential as a therapeutic bacterium for metabolic health.

Short chain fatty acid butyrate, as discussed above, is clearly an important signaling molecule with proven protective effects against several diseases [21,71]. A reduced abundance of butyrate-producing bacteria has been repeatedly observed in type 2 diabetes [72,73,74]. Therefore, it is plausible that butyrate-producing bacteria (either single strains or mixtures) are viable targets for the therapeutic treatment and prevention of metabolic diseases. To date, several butyrate-producing species have shown effects on improving insulin sensitivity in mice and humans. *Anaerobutyricum soehngenii* is the most advanced in the development and is currently in Phase II clinical trials. It was first observed that the level of *E. hallii* (formal nomenclature of *A. soehngenii*) in the small intestine was associated with improved insulin sensitivity after an FMT from lean subjects to those with metabolic syndrome [15]. Similar effects were subsequently observed in an efficacy study in mice [14] and in a mechanistic study in humans [75]. In this mechanistic study, the authors showed that duodenal perfusion of *A. soehngenii* in subjects with metabolic syndrome increased the expression and secretion of GLP1, which was associated with increased fecal butyrate levels. A recent human study showed that the administration of *A. soehngenii* capsules for release in the small intestine improved glycemic controls in metabolic syndrome [43]. Taken together, these data suggest that *A. soehngenii* is a potential therapeutic microbe for metabolic disorders.

*Anaerostipes* is another abundant butyrogenic species that has been associated with reduced metabolic risk in large human cohorts [37,76]. The administration of *Anaerostipes rhamnosivorans* for 6 weeks reduced fasting glucose levels and did not affect insulin sensitivity in diet-induced obese mice [76]. A newly identified butyrate-producing species, *Dysosmobacter welbionis*, was shown to reduce body weight gain and improve glucose metabolism only after at least 9 weeks of supplementation [77]. It is therefore of interest to investigate whether insulin sensitivity and other metabolic parameters can be improved by prolonged oral supplementation with *A. rhamnosivorans*. It has recently been discovered that *Anaerostipes* forms a synergistic interaction with a prevalent gut microbe, *Mitsuokella jalaludinii*, in the conversion of dietary phytate, an abundant bioactive component in plants, to propionate [78]. As plant-based diets and propionate have been associated with metabolic benefits, further studies are awaited to investigate the therapeutic potential of combining these two bacteria with phytate for metabolic health.

There are several species that have been shown to have anti-obesity and anti-diabetic effects. *Christensenella minuta* has been associated with a lean phenotype in a British twin cohort [79]. In the same study, oral supplementation with this bacterium reduced body weight gain and fat mass once administered in obese humanized mice as compared to placebo (obese humanized mice without bacterial supplementation). Reduced levels of *C. minuta* have been observed in subjects with metabolic disorders [80,81], and the levels of this bacterium have been inversely associated with several metabolic risk biomarkers, including BMI, triglycerides and high-density lipoprotein [82]. The observed effects of this bacterium are mostly from mouse studies and no human data have been reported yet. *Intestinimonas butyriciproducens* is another butyrogenic species with a unique metabolism that has the potential to be used as a therapeutic strain for metabolic diseases. It is by far the only gut bacterium that is capable of converting dietary fructoselysine to butyrate [83]. Fructoselysine is an abundant Amadori product formed in cooked foods and is also an intermediate in the formation of advanced glycation end products (AGEs), harmful compounds associated with high cardiometabolic risk [84]. The conversion of fructoselysine to butyrate by *I. butyriciproducens* is highly favorable given the importance of butyrate for metabolic health. This is supported by the observation that the abundance of fructoselysine pathway genes was inversely associated with BMI, fasting insulin and triglycerides in a human cohort, and that 13 weeks of *I. butyriciproducens* administration reduced body weight gain and fat mass and improved insulin sensitivity in a diet-induced obesity mouse model [85]. This was consistent with reduced levels of plasma butyrate in treated mice as compared to placebo. *Parabacteroides distasonis* has been shown to alleviate obesity and metabolic dysfunction in mice via the production of succinate and secondary bile acids [86]. The oral administration of *Odoribacter laneus* was found to improve glucose control and inflammatory profile in obese mice by depleting circulating succinate [87]. Furthermore, supplementation with *Bacteroides thetaiotaomicron* protected mice against obesity in a high-fat-diet-induced obesity model [36]. Engineered bacterial strains have been reported to reprogram the intestinal cells to improve glucose response and insulin secretion [88]. This is an interesting approach with great potential, but the regulation of the use of genetically modified bacteria is strict and requires careful consideration of the use of these strains as therapeutic microbes.

## 6. Microbial Components Affect Host Metabolism

**Figure 4:**
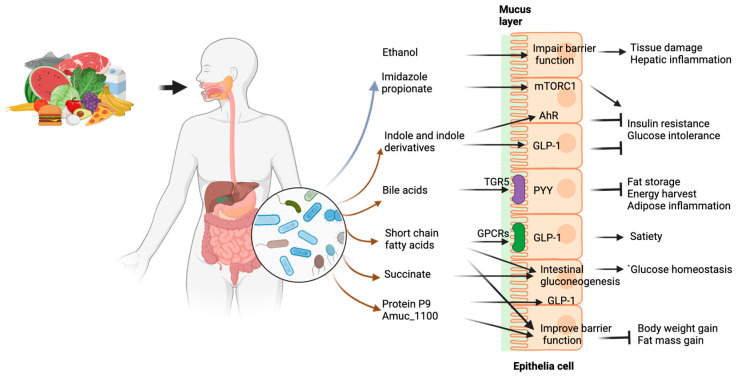
Microbial components regulate host metabolism. Components with a positive influence on host metabolism are indicated by brown arrows, while components with a negative influence are indicated by grey arrows.

Crosstalk between the human microbiota and the host occurs either by direct contact or indirectly through the secretion of components that allow communication between the bacteria and host cells. A number of microbial metabolites have been identified that are associated with the risk of developing diabetes have been identified [89] and many others are associated with obesity, MASLD and T2D [90]. Many newly identified metabolites or components remain to be characterized while the well-studied microbial components in Figure 4 are discussed below.

*Bile acids* are important signaling molecules that have been shown to crosstalk with the human microbiota, thereby affecting host metabolism [91]. Primary bile acids are produced in the liver and released into the duodenum during fat digestion, and the microbiome converts the primary bile acids into secondary bile acids, changes in which have been linked to inflammatory bowel disease [92] and intestinal carcinogenesis [93]. Bile acids have been shown to activate the Takeda G protein-coupled receptor 5 (TGR5), a G-protein-coupled receptor and the farnesoid X receptor FXR, a nuclear hormone receptor; thereby inducing GLP1 release, leading to improved glucose homeostasis [94,95]. Recently, new conjugated bile acids derived from the microbiome have been reported, and these new bile acids have shown activity at the FXR receptor in vitro [96]. A new microbial bile salt hydrolase responsible for acyl transformation has recently been discovered [97], further contributing to the expansion of bile acid diversity. An increased abundance of novel bile acid pathways has been observed in the microbiome of centenarians [98], suggesting that this bile acid synthesis may be associated with longevity. However, the role of these newly identified bile acids or enzymes in health and disease remains to be elucidated.

*Tryptophan* is an essential amino acid that can only be obtained from food. Gut bacteria have been shown to play a central role in tryptophan metabolism, with the ability to convert tryptophan to various molecules, such as indole and its derivatives, thereby contributing to maintain intestinal homeostasis [99]. For example, *Clostridium sporogenes* has been reported to convert tryptophan to both tryptamine and indole-3-propionic acid [100], whereas *Ruminococcus gnavus* only produces tryptamine from the decarboxylation of tryptophan [101]. Indole propionic acid levels have been reported to be associated with a lower risk of developing T2D [102]. In addition, indole-3-propionic acid has been found to regulate intestinal barrier function by interacting with the pregnane X receptor [103] and to improve glucose metabolism in rats [104]. Previous studies have shown that bacterial indole promotes the release of glucagon-like peptide-1 (GLP1) to slow down gastric emptying and reduce the appetite [105]. Indoles produced by the gut microbiota have also been reported to activate the aryl hydrocarbon receptor (AhR) [106], a transcription factor involved in immune regulation and cytokine release, thereby contributing to gut health. As a product of microbial tryptophan metabolism, gut microbiota-derived tryptamine has been shown to impair insulin signaling in animal models by activating the activating MAPK/ERK pathway [107]. A recent study showed that FMT halted the progression of onset type 1 diabetes in which 6-bromo-tryptophan, a metabolite derived from microbial tryptophan metabolism, was associated with prolonged residual ß-cell function [108]. These data suggest that the microbial production of these metabolites may be beneficial for metabolic health. Therapeutic approaches to deliver beneficial tryptophan-derived metabolites have great potential and require validation in mice and humans.

*Short chain fatty acids* (SCFAs) are end metabolites of bacterial fermentation of indigestible dietary components, mainly fiber [21]. They include butyrate, propionate and acetate, which are mainly produced in the caecum and colon in a molar ratio of 1:1:3 [109,110]. SCFAs act as signaling molecules and enter the systemic circulation, thereby affecting the metabolism and function of various peripheral tissues [111]. SCFAs have been shown to activate GPCR receptors (GPR41/43), thereby inducing the secretion of the incretin hormones PYY and GLP1 [112,113], which may contribute to improved glucose metabolism and insulin secretion [114]. SCFAs produced from the fermentation of dietary fiber have been found to activate intestinal gluconeogenesis via a gut–brain neural circuit, providing metabolic benefits for body weight and glucose control [115]. In addition, epithelial barrier function and intestinal permeability were also improved by SCFA exposure through modulation of tight junction protein expression [116,117,118]. Previous studies have shown that maintaining epithelial integrity with low permeability is crucial to prevent leakage of toxic components into the systemic circulation, which can cause chronic inflammation, weight gain and insulin resistance [119,120]. The SCFA butyrate has been shown to interact directly with host receptors to suppress colonic inflammation and carcinogenesis [71]. With all the metabolic benefits observed above, many studies have been conducted to investigate the potential benefits of SCFA supplementation on metabolic health. It has been reported that the oral supplementation of SCFA for 4 weeks improved hepatic metabolic functions via the FFAR3 receptor in mice [121], while 12 weeks of SCFA administration prevented diet-induced obesity in mice through the regulation of GPCR receptors in adipose tissue and colon [122]. The intraperitoneal administration of SCFAs improved lipid metabolism in rats [123]. In addition, mice fed a high-fat diet and treated with tributyrin, a butyrate precursor drug, were protected from diet-induced obesity, insulin resistance and hepatic steatosis [124]. Oral supplementation with butyrate was found to reduce fasting insulin levels in diet-induced obese mice [125]. This is in contrast to a human study in which butyrate supplementation had no beneficial effect on glucose metabolism in subjects with metabolic syndrome [126]. Propionate administration for 22 weeks reduced hepatic lipogenesis and improved insulin sensitivity in a diet-induced obesity mouse model [123]. Two oral supplements of propionate increased resting energy expenditure and lipid oxidation in fasted humans [127]. The discrepancy between animal and human studies warrants the need for well-designed studies to further investigate the benefits of SCFAs in humans. Furthermore, the administration of these short chain fatty acids has produced inconsistent results, which may be due to their rapid absorption in the upper gastrointestinal tract, whereas all SCFA receptors are predominantly located in the lower gastrointestinal tract and colon [128]. While supplementation with SCFAs does not solve the problem of an imbalanced microbiome and must be carried out on a regular basis, the administration of bacterial strains would be more sustainable in this context.

There is an increasing number of microbial metabolites that signal to the host, and *Akkermansia muciniphila* has been shown to improve barrier function and metabolic health in a variety of mouse models [55].The main mode of action has been attributed to its outer membrane protein Amuc_1100, which can bind to Toll-like receptor-2 (TLR-2) to stimulate expression of tight junction proteins and improve barrier function in mice [67]. As this Amuc_1100 protein is thermostable, this rationalizes the similar effect of pasteurized *A. muciniphila* administration in mice. It is noteworthy that a recent human study showed improved barrier function following the administration of pasteurized *A. muciniphila* in obese humans with metabolic syndrome [13]. In addition, a newly identified protein P9 secreted by *A. muciniphila* can bind to ICAM-2 to directly induce the L-cell secretion of GLP1 in mice [68]. The lack of increased GLP1 activity in the human studies suggests that the efficacy in humans remains to be established. Microbial imidazole propionate derived from histidine impaired glucose tolerance and insulin signaling via the activation of mTORC1 [129] and negatively affected metformin action via the inhibition of AMPK activity [130] in mice. Microbiome-derived ethanol has been shown to contribute to the pathogenesis of non-alcoholic fatty liver disease [28]. The oral supplementation of an alcohol-producing strain has been shown to induce fatty liver disease in mice [131]. New approaches to block microbial ethanol production in the gut may offer therapeutic potential in the prevention and treatment of liver disease. In addition, succinate produced by gut bacteria activated intestinal gluconeogenesis, thereby improving glycemic control in mice [132]. Future studies in humans are needed to verify the effects of these newly identified microbial components.

## 7. Conclusions and Future Perspectives

With the rapid increase in the incidence and prevalence of metabolic disorders such as obesity, type 2 diabetes and non-alcoholic fatty liver disease, there is an urgent need for effective therapeutic options to prevent disease progression. It is clear that the human microbiome is strongly associated with metabolic health. There is increasing evidence of causal links between the human microbiome and metabolic disorders in humans and rodents, which holds great promise for the potential use of microbiome-based therapeutics to intervene in host metabolic performance.

Recent advances in sequencing technologies allow in-depth studies of the human microbiome with pathologies of multiple diseases, generating hypotheses for potential bacterial candidates as well as microbial components for disease prevention and treatment. Yet, future research is needed to validate all these hypotheses in well-designed mechanistic and efficacy studies in animal, human and in vitro cell models. The choice of whether to use live or dead bacteria, and the dose of the treatment, will also need to be considered for clinical interventions to achieve optimal benefit. It is certainly necessary to establish more complete reference databases for the identification of novel metabolites or peptides, enabling the discovery of new microbiome-derived components that link the microbiome and host metabolism. Most current human studies rely on the analysis of the fecal microbiome, which does not represent the upper gut microbiome, an important regulator of human health [133]. Therefore, smart sampling capsules with sensing technologies with minimally invasive approaches that allow the measurement of pH, temperature and pressure and collect intestinal samples would be greatly appreciated. It has become increasingly clear that most drugs affect the microbiome and metabolome profiles in addition to their known clinical effects. And perhaps the combination of different bacterial strains with dietary components or drugs can have synergistic effects on host metabolism.

## Abbreviations

FMT	fecal microbiota transplantation
SCFA	short chain fatty acid
BMI	body mass index
T2D	type 2 diabetes
MASLD	metabolic dysfunction-associated liver disease
GLP-1	glucagon-like peptide 1
FFAR3	free fatty acid receptor 3
GPCR	G protein coupled receptors
AMPK	AMP-activated protein kinase
AhR	aryl hydrocarbon receptor
TGR5	takeda G protein-coupled receptor 5
FXR	Farnesoid X receptor FXR
mTORC1	mammalian target of rapamycin complex 1

## References

[1] Rajilić-Stojanović M., de Vos W.M. (2014). The first 1000 cultured species of the human gastrointestinal microbiota. FEMS Microbiol. Rev..

[2] Lynch S.V., Pedersen O. (2016). The Human Intestinal Microbiome in Health and Disease. N. Engl. J. Med..

[3] Yatsunenko T., Rey F.E., Manary M.J., Trehan I., Dominguez-Bello M.G., Contreras M., Magris M., Hidalgo G., Baldassano B.N., Anokhin A.P. (2012). Human gut microbiome viewed across age and geography. Nature.

[4] Qin Y., Havulinna A.S., Liu Y., Jousilahti P., Ritchie S.C., Tokolyi A., Sanders J.G., Valsta L., Brożyńska M., Zhu Q. (2022). Combined effects of host genetics and diet on human gut microbiota and incident disease in a single population cohort. Nat. Genet..

[5] Guarner F., Malagelada J.-R. (2003). Gut flora in health and disease. Lancet.

[6] Chew N.W.S., Ng C.H., Tan D.J.H., Kong G., Lin C., Chin Y.H., Lim W.H., Huang D.Q., Quek J., Fu C.E. (2023). The global burden of metabolic disease: Data from 2000 to 2019. Cell Metab..

[7] Zheng Y., Ley S.H., Hu F.B. (2018). Global aetiology and epidemiology of type 2 diabetes mellitus and its complications. Nat. Rev. Endocrinol..

[8] Jaacks L.M., Vandevijvere S., Pan A., McGowan C.J., Wallace C., Imamura F., Mozaffarian D., Swinburn B., Ezzati M. (2019). The obesity transition: Stages of the global epidemic. Lancet Diabetes Endocrinol..

[9] Younossi Z.M., Koenig A.B., Abdelatif D., Fazel Y., Henry L., Wymer M. (2016). Global epidemiology of nonalcoholic fatty liver disease-Meta-analytic assessment of prevalence, incidence, and outcomes. Hepatology.

[10] Qin J., Li Y., Cai Z., Li S., Zhu J., Zhang F., Liang S., Zhang W., Guan Y., Shen D. (2012). A metagenome-wide association study of gut microbiota in type 2 diabetes. Nature.

[11] Qin N., Yang F., Li A., Prifti E., Chen Y., Shao L., Guo J., Le Chatelier E., Yao J., Wu L. (2014). Alterations of the human gut microbiome in liver cirrhosis. Nature.

[12] Bäckhed F., Ding H., Wang T., Hooper L.V., Koh G.Y., Nagy A., Semenkovich C.F., Gordon J.I. (2004). The gut microbiota as an environmental factor that regulates fat storage. Proc. Natl. Acad. Sci. USA.

[13] Depommier C., Everard A., Druart C., Plovier H., Van Hul M., Vieira-Silva S., Falony G., Raes J., Maiter D., Delzenne N.M. (2019). Supplementation with *Akkermansia muciniphila* in overweight and obese human volunteers: A proof-of-concept exploratory study. Nat. Med..

[14] Udayappan S., Manneras-Holm L., Chaplin-Scott A., Belzer C., Herrema H., Dallinga-Thie G.M., Duncan S.H., Stroes E.S.G., Groen A.K., Flint H.J. (2016). Oral treatment with Eubacterium hallii improves insulin sensitivity in db/db mice. NPJ Biofilms Microbiomes.

[15] Vrieze A., Van Nood E., Holleman F., Salojärvi J., Kootte R.S., Bartelsman J.F.W.M., Dallinga–Thie G.M., Ackermans M.T., Serlie M.J., Oozeer R. (2012). Transfer of Intestinal Microbiota from Lean Donors Increases Insulin Sensitivity in Individuals With Metabolic Syndrome. Gastroenterology.

[16] Asnicar F., Berry S.E., Valdes A.M., Nguyen L.H., Piccinno G., Drew D.A., Leeming E., Gibson R., Le Roy C., Khatib H.A. (2021). Microbiome connections with host metabolism and habitual diet from 1,098 deeply phenotyped individuals. Nat. Med..

[17] Huttenhower C., Gevers D., Knight R., Abubucker S., Badger J.H., Chinwalla A.T., Creasy H.H., Earl A.M., FitzGerald M.G., Fulton R.S. (2012). The Human Microbiome Project Consortium. Structure, function and diversity of the healthy human microbiome. Nature.

[18] Cotillard A., Kennedy S.P., Kong L.C., Prifti E., Pons N., Le Chatelier E., Almeida M., Quinquis B., Levenez F., Galleron N. (2013). Dietary intervention impact on gut microbial gene richness. Nature.

[19] Fujisaka S., Avila-Pacheco J., Soto M., Kostic A., Dreyfuss J.M., Pan H., Ussar S., Altindis E., Li N., Bry L. (2018). Diet, Genetics, and the Gut Microbiome Drive Dynamic Changes in Plasma Metabolites. Cell Rep..

[20] Wastyk H.C., Fragiadakis G.K., Perelman D., Dahan D., Merrill B.D., Yu F.B., Topf M., Gonzalez C.G., Van Treuren W., Han S. (2021). Gut-microbiota-targeted diets modulate human immune status. Cell.

[21] Koh A., De Vadder F., Kovatcheva-Datchary P., Bäckhed F. (2016). From Dietary Fiber to Host Physiology: Short-Chain Fatty Acids as Key Bacterial Metabolites. Cell.

[22] de Vries J., Miller P.E., Verbeke K. (2015). Effects of cereal fiber on bowel function: A systematic review of intervention trials. World J. Gastroenterol..

[23] Roager H.M., Hansen L.B.S., Bahl M.I., Frandsen H.L., Carvalho V., Gøbel R.J., Dalgaard M.D., Plichta D.R., Sparholt M.H., Vestergaard H. (2016). Colonic transit time is related to bacterial metabolism and mucosal turnover in the gut. Nat. Microbiol..

[24] Hu X., Xia K., Dai M., Han X., Yuan P., Liu J., Liu S., Jia F., Chen J., Jiang F. (2023). Intermittent fasting modulates the intestinal microbiota and improves obesity and host energy metabolism. NPJ Biofilms Microbiomes.

[25] Cordova R., Viallon V., Fontvieille E., Peruchet-Noray L., Jansana A., Wagner K.-H., Kyrø C., Tjønneland A., Katzke V., Bajracharya R. (2023). Consumption of ultra-processed foods and risk of multimorbidity of cancer and cardiometabolic diseases: A multinational cohort study. Lancet Reg. Health-Eur..

[26] de Courten B., de Courten M.P., Schalkwijk C.G., Walker K.Z., Forbes J. (2015). Dietary Advanced Glycation End Products Consumption as a Direct Modulator of Insulin Sensitivity in Overweight Humans: A Study Protocol for a Double-Blind, Randomized, Two Period Cross-Over Trial. JMIR Res. Protoc..

[27] Garcia K., Ferreira G., Reis F., Viana S. (2022). Impact of Dietary Sugars on Gut Microbiota and Metabolic Health. Diabetology.

[28] Meijnikman A.S., Davids M., Herrema H., Aydin O., Tremaroli V., Rios-Morales M., Levels H., Bruin S., de Brauw M., Verheij J. (2022). Microbiome-derived ethanol in nonalcoholic fatty liver disease. Nat. Med..

[29] Suez J., Korem T., Zeevi D., Zilberman-Schapira G., Thaiss C.A., Maza O., Israeli D., Zmora N., Gilad S., Weinberger A. (2014). Artificial sweeteners induce glucose intolerance by altering the gut microbiota. Nature.

[30] Ley R., Peterson D., Gordon J. (2006). Ecological and evolutionary forces shaping microbial diversity in the human intestine. Cell.

[31] Zhang C., Zhang M., Wang S., Han R., Cao Y., Hua W., Mao Y., Zhang X., Pang X., Wei C. (2010). Interactions between gut microbiota, host genetics and diet relevant to development of metabolic syndromes in mice. ISME J..

[32] Collado M.C., Isolauri E., Laitinen K., Salminen S. (2008). Distinct composition of gut microbiota during pregnancy in overweight and normal-weight women. Am. J. Clin. Nutr..

[33] Duncan S.H., Lobley G.E., Holtrop G., Ince J., Johnstone A.M., Louis P., Flint H.J. (2008). Human colonic microbiota associated with diet, obesity and weight loss. Int. J. Obes..

[34] Tims S., Derom C., Jonkers D.M., Vlietinck R., Saris W.H., Kleerebezem M., de Vos W.M., Zoetendal E.G. (2013). Microbiota conservation and BMI signatures in adult monozygotic twins. ISME J..

[35] Miller T., Wolin M., de Macario E., Macario A. (1982). Isolation of Methanobrevibacter smithii from human feces. Appl. Environ. Microbiol..

[36] Liu R., Hong J., Xu X., Feng Q., Zhang D., Gu Y., Shi J., Zhao S., Liu W., Wang X. (2017). Gut microbiome and serum metabolome alterations in obesity and after weight-loss intervention. Nat. Med..

[37] Zeevi D., Korem T., Godneva A., Bar N., Kurilshikov A., Lotan-Pompan M., Weinberger A., Fu J., Wijmenga C., Zhernakova A. (2019). Structural variation in the gut microbiome associates with host health. Nature.

[38] Ridaura V.K., Faith J.J., Rey F.E., Cheng J., Duncan A.E., Kau A.L., Griffin N.W., Lombard V., Henrissat B., Bain J.R. (2013). Gut microbiota from twins discordant for obesity modulate metabolism in mice. Science.

[39] Deshpande A.D., Harris-Hayes M., Schootman M. (2008). Epidemiology of diabetes and diabetes-related complications. Phys. Ther..

[40] Le Chatelier E., Nielsen T., Qin J., Prifti E., Hildebrand F., Falony G., Almeida M., Arumugam M., Batto J.-M., Kennedy S. (2013). Richness of human gut microbiome correlates with metabolic markers. Nature.

[41] Karlsson F.H., Tremaroli V., Nookaew I., Bergström G., Behre C.J., Fagerberg B., Nielsen J., Bäckhed F. (2013). Gut metagenome in European women with normal, impaired and diabetic glucose control. Nature.

[42] Attaye I., Witjes J.J., Koopen A.M., van der Vossen E.W.J., Zwirs D., Wortelboer K., Collard D., Kemper E.M., Winkelmeijer M., Holst J.J. (2024). Oral *Anaerobutyricum soehngenii* augments glycemic control in type 2 diabetes. iScience.

[43] Gilijamse P.W., Hartstra A.V., Levin E., Wortelboer K., Serlie M.J., Ackermans M.T., Herrema H., Nederveen A.J., Imangaliyev S., Aalvink S. (2020). Treatment with *Anaerobutyricum soehngenii*: A pilot study of safety and dose–response effects on glucose metabolism in human subjects with metabolic syndrome. NPJ Biofilms Microbiomes.

[44] Wu H., Esteve E., Tremaroli V., Khan M.T., Caesar R., Mannerås-Holm L., Ståhlman M., Olsson L.M., Serino M., Planas-Fèlix M. (2017). Metformin alters the gut microbiome of individuals with treatment-naive type 2 diabetes, contributing to the therapeutic effects of the drug. Nat. Med..

[45] Pryor R., Norvaisas P., Marinos G., Best L., Thingholm L.B., Quintaneiro L.M., De Haes W., Esser D., Waschina S., Lujan C. (2019). Host-Microbe-Drug-Nutrient Screen Identifies Bacterial Effectors of Metformin Therapy. Cell.

[46] Vieira-Silva S., Falony G., Belda E., Nielsen T., Aron-Wisnewsky J., Chakaroun R., Forslund S.K., Assmann K., Valles-Colomer M., Nguyen T.T.D. (2020). Statin therapy is associated with lower prevalence of gut microbiota dysbiosis. Nature.

[47] Schwenger K.J., Clermont-Dejean N., Allard J.P. (2019). The role of the gut microbiome in chronic liver disease: The clinical evidence revised. JHEP Rep..

[48] Jiang W., Wu N., Wang X., Chi Y., Zhang Y., Qiu X., Hu Y., Li J., Liu Y. (2015). Dysbiosis gut microbiota associated with inflammation and impaired mucosal immune function in intestine of humans with non-alcoholic fatty liver disease. Sci. Rep..

[49] Rao R.K., Seth A., Sheth P. (2004). Recent Advances in Alcoholic Liver Disease I. Role of intestinal permeability and endotoxemia in alcoholic liver disease. Am. J. Physiol. Gastrointest. Liver Physiol..

[50] de Medeiros I.C., de Lima J.G. (2015). Is nonalcoholic fatty liver disease an endogenous alcoholic fatty liver disease?—A mechanistic hypothesis. Med. Hypotheses.

[51] Camilleri M., Madsen K., Spiller R., Greenwood-Van Meerveld B., Verne G. (2012). Intestinal barrier function in health and gastrointestinal disease. Neurogastroenterol. Motil..

[52] Thaiss C.A., Levy M., Grosheva I., Zheng D., Soffer E., Blacher E., Braverman S., Tengeler A.C., Barak O., Elazar M. (2018). Hyperglycemia drives intestinal barrier dysfunction and risk for enteric infection. Science.

[53] Benedé-Ubieto R., Cubero F.J., Nevzorova Y.A. (2024). Breaking the barriers: The role of gut homeostasis in Metabolic-Associated Steatotic Liver Disease (MASLD). Gut Microbes.

[54] Winer D.A., Luck H., Tsai S., Winer S. (2016). The Intestinal Immune System in Obesity and Insulin Resistance. Cell Metab..

[55] Segers A., de Vos W.M. (2023). Mode of action of *Akkermansia muciniphila* in the intestinal dialogue: Role of extracellular proteins, metabolites and cell envelope components. Microbiome Res. Rep..

[56] Kootte R.S., Levin E., Salojärvi J., Smits L.P., Hartstra A.V., Udayappan S.D., Hermes G., Bouter K.E., Koopen A.M., Holst J.J. (2017). Improvement of Insulin Sensitivity after Lean Donor Feces in Metabolic Syndrome Is Driven by Baseline Intestinal Microbiota Composition. Cell Metab..

[57] Ha S., Wong V.W.-S., Zhang X., Yu J. (2024). Interplay between gut microbiome, host genetic and epigenetic modifications in MASLD and MASLD-related hepatocellular carcinoma. Gut.

[58] Xue L., Deng Z., Luo W., He X., Chen Y. (2022). Effect of Fecal Microbiota Transplantation on Non-Alcoholic Fatty Liver Disease: A Randomized Clinical Trial. Front. Cell Infect Microbiol..

[59] Merrick B., Allen L., Masirah M.Z.N., Forbes B., Shawcross D.L., Goldenberg S.D. (2020). Regulation, risk and safety of Faecal Microbiota Transplant. Infect Prev. Pract..

[60] van Leeuwen P.T., Brul S., Zhang J., Wortel M.T. (2023). Synthetic microbial communities (SynComs) of the human gut: Design, assembly, and applications. FEMS Microbiol. Rev..

[61] Atarashi K., Tanoue T., Shima T., Imaoka A., Kuwahara T., Momose Y., Cheng G., Yamasaki S., Saito T., Ohba Y. (2011). Induction of colonic regulatory T cells by indigenous Clostridium species. Science.

[62] Douchant K., He S.-M., Noordhof C., Greenlaw J., Vancuren S., Schroeter K., Allen-Vercoe E., Sjaarda C., Vanner S.J., Petrof E.O. (2024). Defined microbial communities and their soluble products protect mice from Clostridioides difficile infection. Commun. Biol..

[63] Collins J., Auchtung J.M. (2017). Control of Clostridium difficile Infection by Defined Microbial Communities. Microbiol. Spectr..

[64] Derrien M., Collado M.C., Ben-Amor K., Salminen S., de Vos W.M. (2008). The mucin degrader *Akkermansia muciniphila* is an abundant resident of the human intestinal tract. Appl. Environ. Microbiol..

[65] Karcher N., Nigro E., Punčochář M., Blanco-Míguez A., Ciciani M., Manghi P., Zolfo M., Cumbo F., Manara S., Golzato D. (2021). Genomic diversity and ecology of human-associated Akkermansia species in the gut microbiome revealed by extensive metagenomic assembly. Genome Biol..

[66] Belzer C., Chia L.W., Aalvink S., Chamlagain B., Piironen V., Knol J., de Vos W.M. (2017). Microbial Metabolic Networks at the Mucus Layer Lead to Diet-Independent Butyrate and Vitamin B(12) Production by Intestinal Symbionts. mBio.

[67] Plovier H., Everard A., Druart C., Depommier C., Van Hul M., Geurts L., Chilloux J., Ottman N., Duparc T., Lichtenstein L. (2017). A purified membrane protein from *Akkermansia muciniphila* or the pasteurized bacterium improves metabolism in obese and diabetic mice. Nat. Med..

[68] Yoon H.S., Cho C.H., Yun M.S., Jang S.J., You H.J., Kim J.-h., Han D., Cha K.H., Moon S.H., Lee K. (2021). *Akkermansia muciniphila* secretes a glucagon-like peptide-1-inducing protein that improves glucose homeostasis and ameliorates metabolic disease in mice. Nat. Microbiol..

[69] Everard A., Belzer C., Geurts L., Ouwerkerk J.P., Druart C., Bindels L.B., Guiot Y., Derrien M., Muccioli G.G., Delzenne N.M. (2013). Cross-talk between *Akkermansia muciniphila* and intestinal epithelium controls diet-induced obesity. Proc. Natl. Acad. Sci. USA.

[70] de la Cuesta-Zuluaga J., Mueller N.T., Corrales-Agudelo V., Velásquez-Mejía E.P., Carmona J.A., Abad J.M., Escobar J.S. (2017). Metformin Is Associated with Higher Relative Abundance of Mucin-Degrading *Akkermansia muciniphila* and Several Short-Chain Fatty Acid-Producing Microbiota in the Gut. Diabetes Care.

[71] Liu P., Wang Y., Yang G., Zhang Q., Meng L., Xin Y., Jiang X. (2021). The role of short-chain fatty acids in intestinal barrier function, inflammation, oxidative stress, and colonic carcinogenesis. Pharmacol. Res..

[72] Vallianou N.G., Stratigou T., Tsagarakis S. (2018). Microbiome and diabetes: Where are we now?. Diabetes Res. Clin. Pract..

[73] Wu H., Tremaroli V., Schmidt C., Lundqvist A., Olsson L.M., Krämer M., Gummesson A., Perkins R., Bergström G., Bäckhed F. (2020). The Gut Microbiota in Prediabetes and Diabetes: A Population-Based Cross-Sectional Study. Cell Metab..

[74] Forslund K., Hildebrand F., Nielsen T., Falony G., Le Chatelier E., Sunagawa S., Prifti E., Vieira-Silva S., Gudmundsdottir V., Krogh Pedersen H. (2015). Disentangling type 2 diabetes and metformin treatment signatures in the human gut microbiota. Nature.

[75] Koopen A., Witjes J., Wortelboer K., Majait S., Prodan A., Levin E., Herrema H., Winkelmeijer M., Aalvink S., Bergman J.J.G.H.M. (2022). Duodenal *Anaerobutyricum soehngenii* infusion stimulates GLP-1 production, ameliorates glycaemic control and beneficially shapes the duodenal transcriptome in metabolic syndrome subjects: A randomised double-blind placebo-controlled cross-over study. Gut.

[76] Bui T.P.N., Mannerås-Holm L., Puschmann R., Wu H., Troise A.D., Nijsse B., Boeren S., Bäckhed F., Fiedler D., deVos W.M. (2021). Conversion of dietary inositol into propionate and acetate by commensal Anaerostipes associates with host health. Nat. Commun..

[77] Le Roy T., Moens de Hase E., Van Hul M., Paquot A., Pelicaen R., Régnier M., Depommier C., Druart C., Everard A., Maiter D. (2021). *Dysosmobacter welbionis* is a newly isolated human commensal bacterium preventing diet-induced obesity and metabolic disorders in mice. Gut.

[78] De Vos W.M., Nguyen Trung M., Davids M., Liu G., Rios-Morales M., Jessen H., Fiedler D., Nieuwdorp M., Bui T.P.N. (2024). Phytate metabolism is mediated by microbial cross-feeding in the gut microbiota. Nat. Microbiol..

[79] Goodrich J.K., Waters J.L., Poole A.C., Sutter J.L., Koren O., Blekhman R., Beaumont M., Van Treuren W., Knight R., Bell J.T. (2014). Human Genetics Shape the Gut Microbiome. Cell.

[80] Lim M.Y., You H.J., Yoon H.S., Kwon B., Lee J.Y., Lee S., Song Y.M., Lee K., Sung J., Ko G. (2017). The effect of heritability and host genetics on the gut microbiota and metabolic syndrome. Gut.

[81] Waters J.L., Ley R.E. (2019). The human gut bacteria Christensenellaceae are widespread, heritable, and associated with health. BMC Biol..

[82] Fu J., Bonder M.J., Cenit M.C., Tigchelaar E.F., Maatman A., Dekens J.A., Brandsma E., Marczynska J., Imhann F., Weersma R.K. (2015). The Gut Microbiome Contributes to a Substantial Proportion of the Variation in Blood Lipids. Circ. Res..

[83] Bui T.P.N., Ritari J., Boeren S., de Waard P., Plugge C.M., de Vos W.M. (2015). Production of butyrate from lysine and the Amadori product fructoselysine by a human gut commensal. Nat. Commun..

[84] Luévano-Contreras C., Gómez-Ojeda A., Macías-Cervantes M.H., Garay-Sevilla M.E. (2017). Dietary Advanced Glycation End Products and Cardiometabolic Risk. Curr. Diabetes Rep..

[85] Rampanelli E., Romp N., Troise A.D., Ananthasabesan J., Wu H., Gül I.S., Pascale S.D., Scaloni A., Bäckhed F., Fogliano V. (2024). Gut bacterium Intestinimonas butyriciproducens improves host metabolic health: Evidence from cohort and animal intervention studies. Research Square.

[86] Wang K., Liao M., Zhou N., Bao L., Ma K., Zheng Z., Wang Y., Liu C., Wang W., Wang J. (2019). Parabacteroides distasonis Alleviates Obesity and Metabolic Dysfunctions via Production of Succinate and Secondary Bile Acids. Cell Rep..

[87] Huber-Ruano I., Calvo E., Mayneris-Perxachs J., Rodríguez-Peña M.M., Ceperuelo-Mallafré V., Cedó L., Núñez-Roa C., Miro-Blanch J., Arnoriaga-Rodríguez M., Balvay A. (2022). Orally administered Odoribacter laneus improves glucose control and inflammatory profile in obese mice by depleting circulating succinate. Microbiome.

[88] Duan F.F., Liu J.H., March J.C. (2015). Engineered commensal bacteria reprogram intestinal cells into glucose-responsive insulin-secreting cells for the treatment of diabetes. Diabetes.

[89] Wang T.J., Larson M.G., Vasan R.S., Cheng S., Rhee E.P., McCabe E., Lewis G.D., Fox C.S., Jacques P.F., Fernandez C. (2011). Metabolite profiles and the risk of developing diabetes. Nat. Med..

[90] Canfora E.E., Meex R.C.R., Venema K., Blaak E.E. (2019). Gut microbial metabolites in obesity, NAFLD and T2DM. Nat. Rev. Endocrinol..

[91] Wahlström A., Sayin S.I., Marschall H.-U., Bäckhed F. (2016). Intestinal Crosstalk between Bile Acids and Microbiota and Its Impact on Host Metabolism. Cell Metab..

[92] Heinken A., Ravcheev D.A., Baldini F., Heirendt L., Fleming R.M.T., Thiele I. (2019). Systematic assessment of secondary bile acid metabolism in gut microbes reveals distinct metabolic capabilities in inflammatory bowel disease. Microbiome.

[93] Cao H., Xu M., Dong W., Deng B., Wang S., Zhang Y., Wang S., Luo S., Wang W., Qi Y. (2017). Secondary bile acid-induced dysbiosis promotes intestinal carcinogenesis. Int. J. Cancer.

[94] Thomas C., Gioiello A., Noriega L., Strehle A., Oury J., Rizzo G., Macchiarulo A., Yamamoto H., Mataki C., Pruzanski M. (2009). TGR5-mediated bile acid sensing controls glucose homeostasis. Cell Metab..

[95] Fiorucci S., Mencarelli A., Palladino G., Cipriani S. (2009). Bile-acid-activated receptors: Targeting TGR5 and farnesoid-X-receptor in lipid and glucose disorders. Trends Pharmacol. Sci..

[96] Quinn R.A., Melnik A.V., Vrbanac A., Fu T., Patras K.A., Christy M.P., Bodai Z., Belda-Ferre P., Tripathi A., Chung L.K. (2020). Global chemical effects of the microbiome include new bile-acid conjugations. Nature.

[97] Guzior D.V., Okros M., Shivel M., Armwald B., Bridges C., Fu Y., Martin C., Schilmiller A.L., Miller W.M., Ziegler K.M. (2024). Bile salt hydrolase acyltransferase activity expands bile acid diversity. Nature.

[98] Sato Y., Atarashi K., Plichta D.R., Arai Y., Sasajima S., Kearney S.M., Suda W., Takeshita K., Sasaki T., Okamoto S. (2021). Novel bile acid biosynthetic pathways are enriched in the microbiome of centenarians. Nature.

[99] Zhang J., Zhu S., Ma N., Johnston L.J., Wu C., Ma X. (2021). Metabolites of microbiota response to tryptophan and intestinal mucosal immunity: A therapeutic target to control intestinal inflammation. Med. Res. Rev..

[100] Dodd D., Spitzer M.H., Van Treuren W., Merrill B.D., Hryckowian A.J., Higginbottom S.K., Le A., Cowan T.M., Nolan G.P., Fischbach M.A. (2017). A gut bacterial pathway metabolizes aromatic amino acids into nine circulating metabolites. Nature.

[101] Williams B.B., Van Benschoten A.H., Cimermancic P., Donia M.S., Zimmermann M., Taketani M., Ishihara A., Kashyap P.C., Fraser J.S., Fischbach M.A. (2014). Discovery and Characterization of Gut Microbiota Decarboxylases that Can Produce the Neurotransmitter Tryptamine. Cell Host Microbe.

[102] de Mello V.D., Paananen J., Lindström J., Lankinen M.A., Shi L., Kuusisto J., Pihlajamäki J., Auriola S., Lehtonen M., Rolandsson O. (2017). Indolepropionic acid and novel lipid metabolites are associated with a lower risk of type 2 diabetes in the Finnish Diabetes Prevention Study. Sci. Rep..

[103] Venkatesh M., Mukherjee S., Wang H., Li H., Sun K., Benechet A.P., Qiu Z., Maher L., Redinbo M.R., Phillips R.S. (2014). Symbiotic Bacterial Metabolites Regulate Gastrointestinal Barrier Function via the Xenobiotic Sensor PXR and Toll-like Receptor 4. Immunity.

[104] Abildgaard A., Elfving B., Hokland M., Wegener G., Lund S. (2018). The microbial metabolite indole-3-propionic acid improves glucose metabolism in rats, but does not affect behaviour. Arch. Physiol. Biochem..

[105] Chimerel C., Emery E., Summers D.K., Keyser U., Gribble F.M., Reimann F. (2014). Bacterial Metabolite Indole Modulates Incretin Secretion from Intestinal Enteroendocrine L Cells. Cell Rep..

[106] Powell D.N., Swimm A., Sonowal R., Bretin A., Gewirtz A.T., Jones R.M., Kalman D. (2020). Indoles from the commensal microbiota act via the AHR and IL-10 to tune the cellular composition of the colonic epithelium during aging. Proc. Natl. Acad. Sci. USA.

[107] Zhai L., Xiao H., Lin C., Wong H.L.X., Lam Y.Y., Gong M., Wu G., Ning Z., Huang C., Zhang Y. (2023). Gut microbiota-derived tryptamine and phenethylamine impair insulin sensitivity in metabolic syndrome and irritable bowel syndrome. Nat. Commun..

[108] de Groot P., Nikolic T., Pellegrini S., Sordi V., Imangaliyev S., Rampanelli E., Hanssen N., Attaye I., Bakker G., Duinkerken G. (2021). Faecal microbiota transplantation halts progression of human new-onset type 1 diabetes in a randomised controlled trial. Gut.

[109] Wong J.M., de Souza R., Kendall C.W., Emam A., Jenkins D.J. (2006). Colonic health: Fermentation and short chain fatty acids. J. Clin. Gastroenterol..

[110] Cummings J.H., Pomare E.W., Branch W.J., Naylor C.P., Macfarlane G.T. (1987). Short chain fatty acids in human large intestine, portal, hepatic and venous blood. Gut.

[111] den Besten G., Bleeker A., Gerding A., van Eunen K., Havinga R., van Dijk T.H., Oosterveer M.H., Jonker J.W., Groen A.K., Reijngoud D.-J. (2015). Short-Chain Fatty Acids Protect Against High-Fat Diet–Induced Obesity via a PPARγ-Dependent Switch from Lipogenesis to Fat Oxidation. Diabetes.

[112] Tolhurst G., Heffron H., Lam Y.S., Parker H.E., Habib A.M., Diakogiannaki E., Cameron J., Grosse J., Reimann F., Gribble F.M. (2012). Short-chain fatty acids stimulate glucagon-like peptide-1 secretion via the G-protein-coupled receptor FFAR2. Diabetes.

[113] Psichas A., Sleeth M.L., Murphy K.G., Brooks L., Bewick G.A., Hanyaloglu A.C., Ghatei M.A., Bloom S.R., Frost G. (2015). The short chain fatty acid propionate stimulates GLP-1 and PYY secretion via free fatty acid receptor 2 in rodents. Int. J. Obes..

[114] Holz G.G.T., Kühtreiber W.M., Habener J.F. (1993). Pancreatic beta-cells are rendered glucose-competent by the insulinotropic hormone glucagon-like peptide-1(7-37). Nature.

[115] De Vadder F., Kovatcheva-Datchary P., Goncalves D., Vinera J., Zitoun C., Duchampt A., Bäckhed F., Mithieux G. (2014). Microbiota-Generated Metabolites Promote Metabolic Benefits via Gut-Brain Neural Circuits. Cell.

[116] Mariadason J.M., Barkla D.H., Gibson P.R. (1997). Effect of short-chain fatty acids on paracellular permeability in Caco-2 intestinal epithelium model. Am. J. Physiol..

[117] Pérez-Reytor D., Puebla C., Karahanian E., García K. (2021). Use of Short-Chain Fatty Acids for the Recovery of the Intestinal Epithelial Barrier Affected by Bacterial Toxins. Front. Physiol..

[118] Suzuki T., Yoshida S., Hara H. (2008). Physiological concentrations of short-chain fatty acids immediately suppress colonic epithelial permeability. Br. J. Nutr..

[119] Cani P.D., Amar J., Iglesias M.A., Poggi M., Knauf C., Bastelica D., Neyrinck A.M., Fava F., Tuohy K.M., Chabo C. (2007). Metabolic endotoxemia initiates obesity and insulin resistance. Diabetes.

[120] Mehta N.N., McGillicuddy F.C., Anderson P.D., Hinkle C.C., Shah R., Pruscino L., Tabita-Martinez J., Sellers K.F., Rickels M.R., Reilly M.P. (2010). Experimental endotoxemia induces adipose inflammation and insulin resistance in humans. Diabetes.

[121] Shimizu H., Masujima Y., Ushiroda C., Mizushima R., Taira S., Ohue-Kitano R., Kimura I. (2019). Dietary short-chain fatty acid intake improves the hepatic metabolic condition via FFAR3. Sci. Rep..

[122] Lu Y., Fan C., Li P., Lu Y., Chang X., Qi K. (2016). Short Chain Fatty Acids Prevent High-fat-diet-induced Obesity in Mice by Regulating G Protein-coupled Receptors and Gut Microbiota. Sci. Rep..

[123] Shah S., Fillier T., Pham T.H., Thomas R., Cheema S.K. (2021). Intraperitoneal Administration of Short-Chain Fatty Acids Improves Lipid Metabolism of Long-Evans Rats in a Sex-Specific Manner. Nutrients.

[124] Vinolo M.A., Rodrigues H.G., Festuccia W.T., Crisma A.R., Alves V.S., Martins A.R., Amaral C.L., Fiamoncini J., Hirabara S.M., Sato F.T. (2012). Tributyrin attenuates obesity-associated inflammation and insulin resistance in high-fat-fed mice. Am. J. Physiol. Endocrinol. Metab..

[125] Mollica M.P., Mattace Raso G., Cavaliere G., Trinchese G., De Filippo C., Aceto S., Prisco M., Pirozzi C., Di Guida F., Lama A. (2017). Butyrate Regulates Liver Mitochondrial Function, Efficiency, and Dynamics in Insulin-Resistant Obese Mice. Diabetes.

[126] Bouter K.E., Bakker G.J., Levin E., Hartstra A.V., Kootte R.S., Udayappan S.D., Katiraei S., Bahler L., Gilijamse P.W., Tremaroli V. (2018). Differential metabolic effects of oral butyrate treatment in lean versus metabolic syndrome subjects. Clin. Transl. Gastroenterol..

[127] Chambers E.S., Byrne C.S., Aspey K., Chen Y., Khan S., Morrison D.J., Frost G. (2018). Acute oral sodium propionate supplementation raises resting energy expenditure and lipid oxidation in fasted humans. Diabetes Obes. Metab..

[128] Suzuki K., Iwasaki K., Murata Y., Harada N., Yamane S., Hamasaki A., Shibue K., Joo E., Sankoda A., Fujiwara Y. (2018). Distribution and hormonal characterization of primary murine L cells throughout the gastrointestinal tract. J. Diabetes Investig..

[129] Koh A., Molinaro A., Ståhlman M., Khan M.T., Schmidt C., Mannerås-Holm L., Wu H., Carreras A., Jeong H., Olofsson L.E. (2018). Microbially Produced Imidazole Propionate Impairs Insulin Signaling through mTORC1. Cell.

[130] Koh A., Mannerås-Holm L., Yunn N.-O., Nilsson P.M., Ryu S.H., Molinaro A., Perkins R., Smith J.G., Bäckhed F. (2020). Microbial Imidazole Propionate Affects Responses to Metformin through p38γ-Dependent Inhibitory AMPK Phosphorylation. Cell Metab..

[131] Yuan J., Chen C., Cui J., Lu J., Yan C., Wei X., Zhao X., Li N., Li S., Xue G. (2019). Fatty Liver Disease Caused by High-Alcohol-Producing Klebsiella pneumoniae. Cell Metab..

[132] De Vadder F., Kovatcheva-Datchary P., Zitoun C., Duchampt A., Bäckhed F., Mithieux G. (2016). Microbiota-Produced Succinate Improves Glucose Homeostasis via Intestinal Gluconeogenesis. Cell Metab..

[133] Ruigrok R., Weersma R.K., Vila A.V. (2023). The emerging role of the small intestinal microbiota in human health and disease. Gut Microbes.

